# Tin Fractured Splints

**Published:** 1861-09

**Authors:** 


Tin Fracture Splints.—For a long time, we have been in the habit of using tin instead of wood for fracture splints. This material is so far superior to all others used for the purpose, that we are greatly surprised that manufacturers of patented splints have never adopted it instead of wood. It is lighter, stronger, far cheaper, and can be moulded into any desirable shape by the surgeon. Thus, as often the case in compound fractures, it being desirable to have some particular part exposed, the surgeon may go to any tinner, and procure, at very trifling expense, the splints, with the necessary openings, which cannot be the case with the use of wood. *Any* tinman can easily make the splints, but few workmen can make them properly shaped of wood. We would particularly commend them to young practitioners, who may not be able to equip themselves with the more costly appliances, in the commencement of their practical careers.—*San Francisco Medical Press.*

